# Phytochemistry and Pharmacology of Sesquiterpenoids from *Atractylodes* DC. Genus Rhizomes

**DOI:** 10.3390/molecules29061379

**Published:** 2024-03-20

**Authors:** Zhengyi Qu, Hongqun Liu, Zhenghai Zhang, Peihe Zheng, Shuting Zhao, Wei Hou

**Affiliations:** 1 Institute of Special Wild Economic Animals and Plants, Chinese Academy of Agricultural Sciences, Changchun 130112, China; qu9581@163.com (Z.Q.);; 2 Jilin Provincial Key Laboratory of Traditional Chinese Medicinal Materials Cultivation and Propagation, Changchun 130112, China; 3 Medical School, Changchun Sci-Tech University, Changchun 130600, China; 4 Traditional Chinese Medicine School, Jilin Agricultural Science and Technology University, Jilin 132109, China

**Keywords:** *Atractylodes* DC. rhizomes, sesquiterpenes, eudesmane-type, phytochemistry, biosynthetic pathways, pharmacology

## Abstract

The rhizomes of the genus *Atractylodes* DC. consist of various bioactive components, including sesquiterpenes, which have attracted a great deal of research interest in recent years. In the present study, we reviewed the previously published literatures prior to November 2023 on the chemical structures, biosynthetic pathways, and pharmacological activities of the sesquiterpenoids from this genus via online databases such as Web of Science, Google Scholar, and ScienceDirect. Phytochemical studies have led to the identification of more than 160 sesquiterpenes, notably eudesmane-type sesquiterpenes. Many pharmacological activities have been demonstrated, particularly anticancer, anti-inflammatory, and antibacterial and antiviral activities. This review presents updated, comprehensive and categorized information on the phytochemistry and pharmacology of sesquiterpenes in *Atractylodes* DC., with the aim of offering guidance for the future exploitation and utilization of active ingredients in this genus.

## 1. Introduction

The genus *Atractylodes* DC. belongs to the family Asteraceae and mainly is distributed in Eastern Asia [1]. There are four accepted names for this genus according to World Flora Online: *Atractylodes lancea* DC. (*A. lancea*), *Atractylodes macrocephala* Koidz. (*A. macrocephala*), *Atractylodes carlinoides* (Hand.-Mazz.) Kitam. (*A. carlinoides*), and *Atractylodes koreana* (Nakai) Kitam. (*A. koreana*). Moreover, 60 synonyms of *A. lancea* are listed in this revision, such as *Atractylodes japonica*, *Atractylodes chinensis*, and *Atractylodes ovata* [2].

The rhizomes of the *Atractylodes* DC. genus are rich in essential oils, which have been traditionally used for the treatment of gastrointestinal, coronavirus, and rheumatic diseases in China, Korea, and Japan [3,4,5,6]. The rhizomes of *A. lancea* have been used as crude drugs in the Chinese and Japanese pharmacopoeia, which are referred to Cangzhu and Sojutsu, respectively [7,8]. In addition, *A. lancea* (known as Khod-Kha-Mao in Thailand) is also used for the treatment of fevers and colds in Thai traditional medicine [9]. *A. macrocephala* is not only used as functional food in China but has also been historically widely used in traditional Korean and Japanese medicine [10]. These traditional uses of *Atractylodes* DC. are closely related to its intrinsic chemical composition [11,12].

Sesquiterpenes are significant oily compositions with extensive dispersal in plants, currently gaining recognition due to their wide range of pharmacological effects, including antitumor, anti-inflammatory, antibacterial and antiviral, etc. [13,14,15]. According to the diverse skeletal structures of sesquiterpenes in *Atractylodes* DC., they can be divided into the following five categories: eudesmane-type (such as *β*-eudesmol, atractylon), guaiane-type (such as atractylmacrol A, atrchiterpene D), spirovetivane-type (such as hinesol, hinesolone), isopterocarpolone-type (such as 14-hydroxy-isopterocarpolone, Atractyloside I), and eremophilane-type [10,16,17,18,19,20,21]. *β*-eudesmol, atractylon, and hinesol are usually used as chemical markers for evaluating the quality of *Atractylodes* DC. in different regions [22,23,24,25]. Many studies have been conducted on *Atractylodes* DC. [26,27], yet there are still noticeable deficiencies in the literature. Various potential clinical uses and upcoming research paths have been suggested, offering a comprehensive collection of research discoveries on the sesquiterpenes of *Atractylodes* DC. Hence, the current work presents the chemical constituents, possible biosynthesis, and pharmacologic mechanisms of sesquiterpenoids from *Atractylodes* DC. genus rhizomes in order to encourage researchers to explore this genus in depth with the aim of discovering novel bioactive substances.

## 2. Methodology

This present review article considered the previously published literature prior to November 2023 concerning the chemical components, biosynthetic pathways, and pharmacological activities of sesquiterpenoids from the genus *Atractylodes* DC. The search was conducted using online databases such as Web of Science, Google Scholar, ScienceDirect, PubMed, CNKI, Baidu Scholar, and classic books on Dictionary of TCM. The key words searched included *Atractylodes* DC., Asteraceae, secondary metabolites, phytochemistry, sesquiterpenoids, biosynthetic, atractylenolides, biological activity, pharmacological, and the names of each species of the genus. The chemical structures were drawn using ChemDraw Professional 14.0 software.

## 3. Phytochemical Constituents

Our literature investigation revealed that essential oils are the main active ingredient in the genus *Atractylodes*, among which sesquiterpenoids are the characteristic components. Currently, 163 sesquiterpenoids have been isolated and identified from the genus *Atractylodes* DC., including 104 eudesmane-type, 32 guaiane-type, 14 spirovetivane-type, 11 isopterocarpolone-type, and 2 eremophilane-type sesquiterpenoids. Their specific chemical names, structures, sources, collection areas, and year of isolation are shown in Table 1, Table 2, Table 3, Table 4 and Table 5.

### 3.1. Eudesmane-Type Sesquiterpenes

This group of sesquiterpenoids possesses a 5,8*α*-dimethyl-3-(propan-2-yl)-decahydronaphthalene skeleton, which is abundant in the *Atractylodes* DC. genus [28,29,30]. Among them, compounds (**1**–**5**, **11**–**13**, **28**, **34**, **39, 42**, **61**, **68**, **77**, **78**, **82**, **83**, **85**, **87**, **89**, **90**–**96**, **98**) have eudesmane lactone structures, compounds (**21**, **22**, **36**, **37**, **41**, **75**, **76**, **79**, **80**, **97**) are *N*-containing eudesmanes, and compounds (**18**–**20**, **38**, **40**) possess epoxy ring groups. Atractylenolides (I–III) have lactone structures and possess antioxidant, anti-inflammatory, and anticancer properties [31]. Atractylenolide I (AT-I) (**1**), atractylenolide II (AT-II) (**2**), atractylenolide III (AT-III) (**3**), and atractylenolide IV (**4**) are widely present in *A. lancea* and *A. macrocephala* [32,33,34,35,36,37]. Atractylenolide V (**5**), atractylenolide VI (**6**), atractylenolide VII (**7**), and biatractylenolide II (**8**) have been reported in *A. macrocephala* [38,39,40,41]. A phytochemical investigation of *A. macrocephala* 95% ethanol extract identified five eudesmane-type sesquiterpenoids (**9**–**13**), and their structures were elucidated via NMR and high-resolution electrospray ionization mass spectroscopy (HRESIMS) analyses, X-ray diffraction analyses, and electronic circular dichroism (ECD) [18]. Four new sesquiterpenoids eudesm-4(15),7-diene-3*α*,9*β*,11-triol (**14**), and eudesm-4(15),7-diene-1*β*,3*α*,9*β*,11-tetraol (**15**), (7*Z*)-8*β*,13-diacetoxy-eudesma-4(15),7(11)-diene (**16**), 7-oxo-7,8-secoeudesma-4(15),11-dien-8-oic acid (**17**), were purified from the ethanol extract of *A. macrocephala* using column chromatography on silica gel, Sephadex LH-20, ODS, and high-performance liquid chromatography (HPLC) [42,43]. Zhang et al. [44] identified atramacronoids A–C (**18**–**20**) from the rhizomes of *A. macrocephala* using spectroscopic data analysis, chemical calculations, and X-ray diffraction, which were found to contain an unusual 6/6/5/5/6 skeleton furnished by an unexpected C-8–C-16 linkage. Subsequently, twenty undescribed eudesmane-type sesquiterpenes named atramacronoids D–W (**21**–**40**) were identified in the rhizomes of *A. macrocephala* using extensive spectroscopic data analysis, Snatzke’s rule, ECD calculations, and X-ray crystallographic analysis [45,46]. A chemical investigation of the ethanol extract of *A. lancea* resulted in the isolation of nine eudesmane-type sesquiterpenoids (**41**–**49**), and their structures were elucidated using spectroscopic techniques and HRESIMS [47]. Kamauchi et al. [48] gained two new eudesmane-type sesquiterpenoids, namely 3*α*-hydroxy pterocarpol (**50**) and (11*R*)-2,11,12-trihydroxy-b-selinene (**51**), along with three known sesquiterpenoids (**52**–**54**) in the fermented rhizomes of *A. lancea* using column chromatography. *β*-eudesmol (**55**) and atractylon (**98**) were widely distributed in genus species [26,49,50,51,52]. (1*R*,7*R*,10*R*)-1-hydroxylcarissone-11-*O*-*β*-d-glucopyranoside (**56**) was isolated from *A. lancea* via HPLC and elucidated through detailed spectroscopic methods [53]. A phytochemical investigation of the rhizomes of *A. macrocephala* led to the isolation of four new sesquiterpenes, atractylmacrols B–E (**57**–**60**), as well as known eudesmane sesquiterpenes (**61**) through the interpretation of their NMR spectroscopic data and HREIMS values [54]. Eight eudesmane-type sesquiterpenoids (**62**–**69**) were previously isolated from *A. lancea* with normal-phase and reverse-phase column chromatography and elucidated through detailed spectroscopic methods [55,56]. Xu et al. [57] identified (2*S*,7*R*,10*S*)-3-hydroxylcarissone-11-*O*-*β*-d-glucopyranoside (**70**) and (2*R*,7*R*,10*S*)-3-hydroxylcarissone-11-*O*-*β*-d-glucopyranoside (**71**) in the rhizomes of *A. lancea* using extensive spectroscopic analyses with experimental and ECD calculations. Eudesm-4(15)-ene-7*α*,11-diol (**72**), (5*R*,10*S*)-eudesm-4(15),7-diene-11-ol-9-one (**73**), and eudesm-4(15),7(11)-diene-9*α*,11-diol (**74**) were separated from *A. lancea* via silica gel column chromatography and preparative TLC [58]. Two new nitrogen-containing sesquiterpenoids, atractylenolactam A (**75**) and atractylenolactam B (**76**); two new sesquiterpene lactones, 8-methoxy-AT-V (**77**) and 15-acetoxyl AT-III (**86**); and four known analogs (**78**–**82**) were separated from *A. macrocephala* using column chromatography and preparative HPLC, and the absolute configurations were established using time-dependent density functional theory ECD (TDDFT-ECD) calculations [59,60]. Zhou et al. [61] isolated six eudesmane-type sesquiterpenoids (**83**–**88**) from *A. lancea* with repeated silica gel column chromatography, and their structures were determined using physiochemical and spectroscopic evidence. Nine atractylenolides (**89**–**97**) with lactone structures were isolated from *A. macrocephala* using silica gel, ODS column chromatography, and preparative HPLC [18,32,35,38,39,62]. Toda et al. [63] purified eudesma-4(14),7(11)-dien-8-one (**100**) from *A. lancea* using silica gel column chromatography and preparative TLC, identified using physiochemical and spectroscopic evidence. Three new eudesmane-type sesquiterpenoids, selina-4(14),7,11-trien-9-ol (**101**), selina-4(14),11-dien-7-ol (**102**), and atractin A (**103**), along with two known compounds, eudesm-4(15)-ene-7*β*,11-diol (**99**) and selina-4(14),7-dien-11-ol (**104**), were separated from *A. macrocephala* using silica gel column chromatography and preparative HPLC, combined with HRESIMS, extensive spectroscopic data, and ECD [28,64]. The eudesmane-type sesquiterpenoids from genus *Atractylodes* DC. are shown in Table 1.

**Table 1:** Eudesmane-type sesquiterpenoids from genus *Atractylodes* DC.

NO.	Compounds	Structure	Source	Collection Area	Year
1	Atractylenolide I	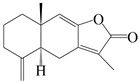	*A. macrocephala* [32], *A. lancea* [33]	China (Yuqian town, Zhejiang province); China (Heilongjiang province)	2017, 2010
2	Atractylenolide II	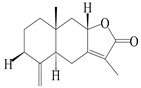	*A. macrocephala* [32], *A. lancea* [34]	China (Yuqian town, Zhejiang province); Germany (Hospital for Traditional Chinese Medicine, Kötzting)	2017, 1998
3	Atractylenolide III/codonolactone	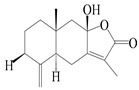	*A. macrocephala* [32], *A. lancea* [35]	China (Yuqian town, Zhejiang province); Japan (Kampo Research Laboratories, Kracie Pharma, Ltd., Takaoka)	2017, 2016
4	Atractylenolide IV	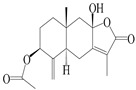	*A. macrocephala* [39], *A. lancea* [36]	China (Pan’an county, Zhejiang province); China (Maoshan mountain of Jiangsu province)	2014, 2008
5	Atractylenolide V	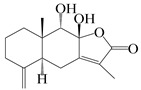	*A. macrocephala* [38]	Korea (Ulsan-si Market)	2016
6	Atractylenolide VI	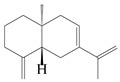	*A. macrocephala* [40]	–	2005
7	Atractylenolide VII	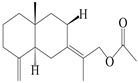	*A. macrocephala* [40]	–	2005
8	Biatractylenolide II	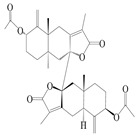	*A. macrocephala* [41]	China (Qimen county, Anhui province)	2017
9	(1*S*,5*R*,10*S*)-atractylmacrene C	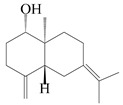	*A. macrocephala* [18]	China (Bozhou Medicinal Materials Market)	2021
10	(1*R*,5*S*,10*R*)-atractylmacrene C	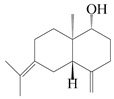	*A. macrocephala* [18]	China (Bozhou Medicinal Materials Market)	2021
11	4*R*,5*R*,8*S*,9*S*-diepoxyatractylenolide II	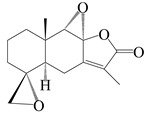	*A. macrocephala* [18]	China (Bozhou Medicinal Materials Market)	2021
12	4-oxo-8*S*,9*S*-epoxylatractylenolide II	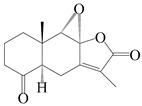	*A. macrocephala* [18]	China (Bozhou Medicinal Materials Market)	2021
13	8*S*,9*S*-epoxylatractylenolide II	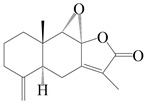	*A. macrocephala* [18]	China (Bozhou Medicinal Materials Market)	2021
14	Eudesm-4(15),7-diene-3*α*,9*β*,11-triol	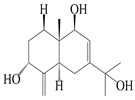	*A. macrocephala* [42]	Vietnam (Quan ba city, Ha Giang province)	2023
15	Eudesm-4(15),7-diene-1*β*,3*α*,9*β*,11-tetraol	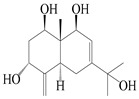	*A. macrocephala* [42]	Vietnam (Quan ba city, Ha Giang province)	2023
16	(7*Z*)-8*β*,13-diacetoxy-eudesma-4(15),7(11)-diene	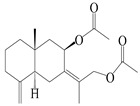	*A. macrocephala* [43]	China (Jiaozuo city, Henan province)	2022
17	7-oxo-7,8-secoeudesma-4(15),11-dien-8-oic acid	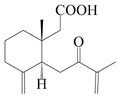	*A. macrocephala* [43]	China (Jiaozuo city, Henan province)	2022
18	Atramacronoid A	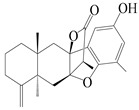	*A. macrocephala* [44]	China (Bozhou Medicinal Materials Market)	2023
19	Atramacronoid B	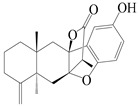	*A. macrocephala* [44]	China (Bozhou Medicinal Materials Market)	2023
20	Atramacronoid C	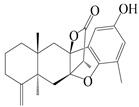	*A. macrocephala* [44]	China (Bozhou Medicinal Materials Market)	2023
21	Atramacronoid D	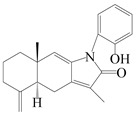	*A. macrocephala* [45]	China (Bozhou Medicinal Materials Market)	2023
22	Atramacronoid E	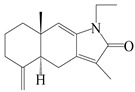	*A. macrocephala* [45]	China (Bozhou Medicinal Materials Market)	2023
23	Atramacronoid F	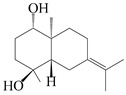	*A. macrocephala* [45]	China (Bozhou Medicinal Materials Market)	2023
24	Atramacronoid G	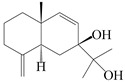	*A. macrocephala* [45]	China (Bozhou Medicinal Materials Market)	2023
25	Atramacronoid H	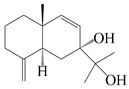	*A. macrocephala* [45]	China (Bozhou Medicinal Materials Market)	2023
26	Atramacronoid I	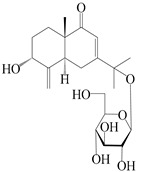	*A. macrocephala* [45]	China (Bozhou Medicinal Materials Market)	2023
27	Atramacronoid J	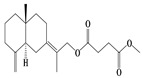	*A. macrocephala* [45]	China (Bozhou Medicinal Materials Market)	2023
28	Atramacronoid K	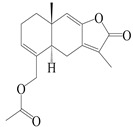	*A. macrocephala* [45]	China (Bozhou Medicinal Materials Market)	2023
29	Atramacronoid L	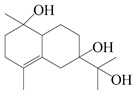	*A. macrocephala* [45]	China (Bozhou Medicinal Materials Market)	2023
30	Atramacronoid M	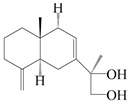	*A. macrocephala* [45]	China (Bozhou Medicinal Materials Market)	2023
31	Atramacronoid N	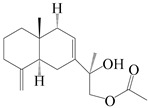	*A. macrocephala* [45]	China (Bozhou Medicinal Materials Market)	2023
32	Atramacronoid O	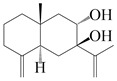	*A. macrocephala* [45]	China (Bozhou Medicinal Materials Market)	2023
33	Atramacronoid P	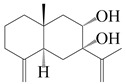	*A. macrocephala* [45]	China (Bozhou Medicinal Materials Market)	2023
34	Atramacronoid Q	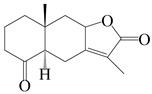	*A. macrocephala* [45]	China (Bozhou Medicinal Materials Market)	2023
35	Atramacronoid R	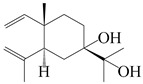	*A. macrocephala* [45]	China (Bozhou Medicinal Materials Market)	2023
36	Atramacronoid S	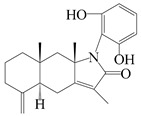	*A. macrocephala* [46]	China (Bozhou Medicinal Materials Market)	2023
37	Atramacronoid T	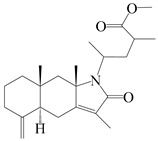	*A. macrocephala* [46]	China (Bozhou Medicinal Materials Market)	2023
38	Atramacronoid U	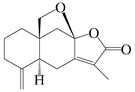	*A. macrocephala* [46]	China (Bozhou Medicinal Materials Market)	2023
39	Atramacronoid V	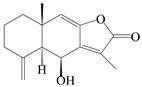	*A. macrocephala* [46]	China (Bozhou Medicinal Materials Market)	2023
40	Atramacronoid W	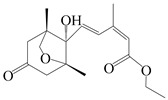	*A. macrocephala* [46]	China (Bozhou Medicinal Materials Market)	2023
41	Atrchiterpene A	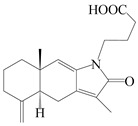	*A. lancea* [47]	China (Heilongjiang province)	2022
42	Atrchiterpene B	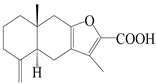	*A. lancea* [47]	China (Heilongjiang province)	2022
43	Atrchiterpene C	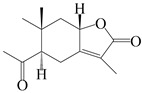	*A. lancea* [47]	China (Heilongjiang province)	2022
44	4(15)-eudesmene-1*β*,7,11-triol	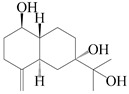	*A. lancea* [47]	China (Heilongjiang province)	2022
45	3-eudesmene-1*β*,7,11-triol	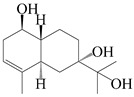	*A. lancea* [47]	China (Heilongjiang province)	2022
46	Eudesmane-4*α*,11,15-triol	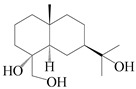	*A. lancea* [39]	China (Heilongjiang province)	2022
47	(4*α*,7*β*,9*α*)-farfugane-4,9,11-triol	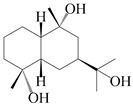	*A. lancea* [47]	China (Heilongjiang province)	2022
48	(4*α*,7*α*,9*α*)-farfugane-4,9,11-triol	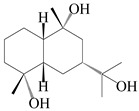	*A. lancea* [47]	China (Heilongjiang province)	2022
49	(1*β*,4*α*,6*β*)-gorgonane-1*β*,4*α*,11-triol	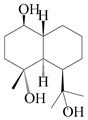	*A. lancea* [47]	China (Heilongjiang province)	2022
50	3*α*-hydroxy pterocarpol	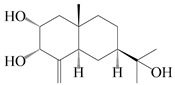	*A. lancea* [48]	Japan (Tokyo city, Kinokuniyakanyakkyoku. Co., Ltd.)	2015
51	(11*R*)-2,11,12-trihydroxy-*β*-selinene	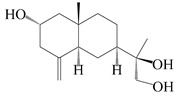	*A. lancea* [48]	Japan (Tokyo city, Kinokuniyakanyakkyoku. Co., Ltd.)	2015
52	Pterocarpol	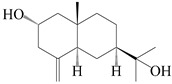	*A. lancea* [48]	Japan (Tokyo city, Kinokuniyakanyakkyoku. Co., Ltd.)	2015
53	Kudtdiol	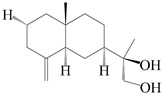	*A. lancea* [48]	Japan (Tokyo city, Kinokuniyakanyakkyoku. Co., Ltd.)	2015
54	(11*S*)-2,11,13-trihydroxy-*β*-selinene	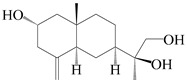	*A. lancea* [48]	Japan (Tokyo city, Kinokuniyakanyakkyoku. Co., Ltd.)	2015
55	*β*-Eudesmol	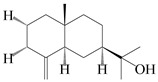	*A. macrocephala* [50], *A. lancea* [49]	China (Qimen city); China (Anguo Chinese Herbs Market, Hebei province)	2021, 2011
56	(1*R*,7*R*,10*R*)-1-hydroxylcarissone-11-*O*-*β*-d-glucopyranoside	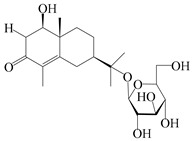	*A. lancea* [53]	China (Huanggang city, Hubei province)	2018
57	Atractylmacrol B	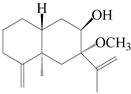	*A. macrocephala* [54]	China (Juhuacun Chinese Traditional Medicine Market, Kunming city, Yunnan province)	2018
58	Atractylmacrol C	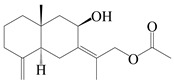	*A. macrocephala* [54]	China (Juhuacun Chinese Traditional Medicine Market, Kunming city, Yunnan province)	2018
59	Atractylmacrol D	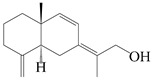	*A. macrocephala* [54]	China (Juhuacun Chinese Traditional Medicine Market, Kunming city, Yunnan province)	2018
60	Atractylmacrol E	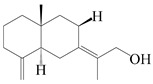	*A. macrocephala* [54]	China (Juhuacun Chinese Traditional Medicine Market, Kunming city, Yunnan province)	2018
61	8*β*-methoxy-atractylenolide I	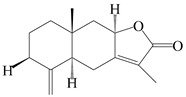	*A. macrocephala* [54]	China (Juhuacun Chinese Traditional Medicine Market, Kunming city, Yunnan province)	2018
62	(3*S*)-3-hydroxyatractylenolide III 3-O-*β*-d-glucopyranoside	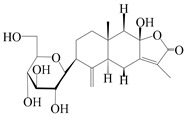	*A. lancea* [55]	Japan (Tokyo city, Metropolitan Medical Plants Garden)	2003
63	Atractyloside C	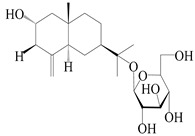	*A. lancea* [56]	Japan (Tokyo city, Metropolitan Medical Plants Garden)	1989
64	Atractyloside D	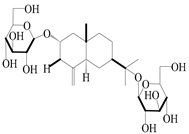	*A. lancea* [56]	Japan (Tokyo city, Metropolitan Medical Plants Garden)	1989
65	Atractyloside E	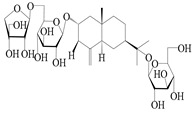	*A. lancea* [56]	Japan (Tokyo city, Metropolitan Medical Plants Garden)	1989
66	Atractyloside F	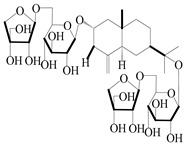	*A. lancea* [56]	Japan (Tokyo city, Metropolitan Medical Plants Garden)	1989
67	Atractyloside G	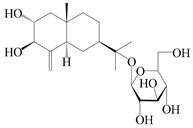	*A. lancea* [56]	Japan (Tokyo city, Metropolitan Medical Plants Garden)	1989
68	Atractyloside H	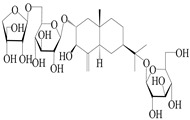	*A. lancea* [56]	Japan (Tokyo city, Metropolitan Medical Plants Garden)	1989
69	Atractyloside G 2-*O*-*β*-d-glucopyranoside	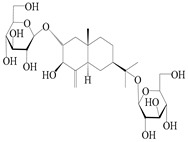	*A. lancea* [56]	Japan (Tokyo city, Metropolitan Medical Plants Garden)	1989
70	(2S,7*R*,10*S*)-3-hydroxylcarissone-11-*O*-*β*-d-glucopyranoside	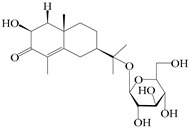	*A. lancea* [57]	China (Huanggang city, Hubei province)	2016
71	(2*R*,7*R*,10*S*)-3-hydroxylcarissone-11-*O*-*β*-d-glucopyranoside	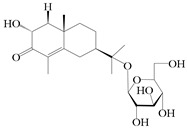	*A. lancea* [57]	China (Huanggang city, Hubei province)	2016
72	Eudesm-4(15)-ene-7*α*,11-diol	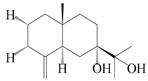	*A. lancea* [58]	China (Lanzhou city, Gansu province)	2008
73	(5*R*,10*S*)-Eudesm-4(15),7-diene-11-ol-9-one	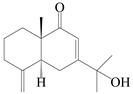	*A. lancea* [58]	China (Lanzhou city, Gansu province)	2008
74	Eudesm-4(15),7(11)-diene-9*α*,11-diol	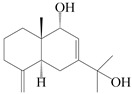	*A. macrocephala* [37], *A. lancea* [58]	China (Hangzhou city, Zhejiang province); China (Lanzhou city, Gansu province)	2011, 2008
75	Atractylenolactam A	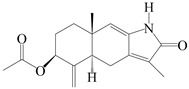	*A. macrocephala* [59]	China (Jiaozuo city, Henan province)	2022
76	Atractylenolactam B	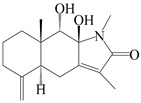	*A. macrocephala* [59]	China (Jiaozuo city, Henan province)	2022
77	8-methoxy-atractylenolide V	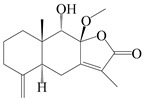	*A. macrocephala* [59]	China (Jiaozuo city, Henan province)	2022
78	15-acetoxyl atractylenolide III	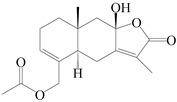	*A. macrocephala* [59]	China (Jiaozuo city, Henan province)	2022
79	Taenialactam A	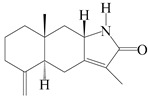	*A. macrocephala* [59]	China (Jiaozuo city, Henan province)	2022
80	Taenialactam B	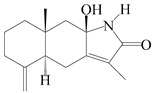	*A. macrocephala* [59]	China (Jiaozuo city, Henan province)	2022
81	Eudesma-4(15),7(11)-dien-8-one	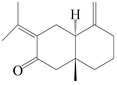	*A. macrocephala* [60]	China (Zhejiang province)	1987
82	8*β*-methoxyatractylenolide	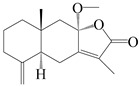	*A. macrocephala* [60]	China (Zhejiang province)	1987
83	4*R*,15-epoxyatractylenolide II	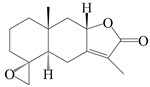	*A. macrocephala* [52], *A. lancea* [61]	China (Pan’an county, Zhejiang province); China (Haerbin city, Heilongjiang province)	2018, 2020
84	Eudesma-7(11)-en-4-ol	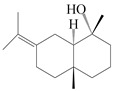	*A. macrocephala* [54], *A. lancea* [61]	China (Juhuacun Chinese Traditional Medicine Market, Kunming city, Yunnan province); China (Haerbin city, Heilongjiang province)	2018, 2020
85	8*β*,9*α*-dihydroxyatractylenolide II	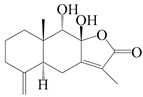	*A. macrocephala* [52], *A. lancea* [61]	China (Pan’an county, Zhejiang province); China (Haerbin city, Heilongjiang province)	2018, 2020
86	Biepiasterolide	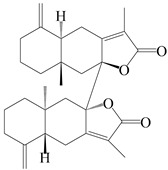	*A. lancea* [61]	China (Haerbin city, Heilongjiang province)	2020
87	Atractylenother	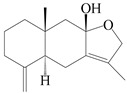	*A. macrocephala* [39], *A. lancea* [61]	China (Pan’an county, Zhejiang province); China (Haerbin city, Heilongjiang province)	2014, 2020
88	Biatractylenolide	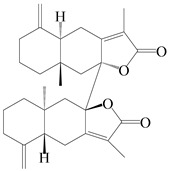	*A. lancea* [61]	China (Haerbin city, Heilongjiang province)	2020
89	Isoatractylenolide I	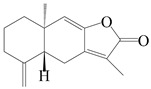	*A. macrocephala* [32], *A. lancea* [61]	China (Yuqian town, Zhejiang province); China (Haerbin city, Heilongjiang province)	2017, 2020
90	3*β*-acetoxyl atractylenolide I	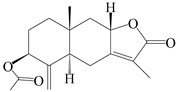	*A. macrocephala* [32]	China (Yuqian town, Zhejiang province)	2017
91	4*R*,15-epoxy-8*β*-hydroxyatractylenolide II	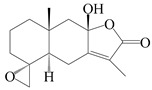	*A. macrocephala* [39]	China (Pan’an county, Zhejiang Province)	2014
92	8-epiatractylenolide III	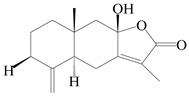	*A. macrocephala* [39]	China (Pan’an county, Zhejiang Province)	2014
93	8-epiasterolid	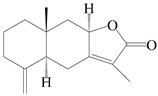	*A. macrocephala* [18], *A. lancea* [35]	China (Bozhou Medicinal Materials Market); Kampo Research Laborato-ries, Kracie Pharma, Ltd., Ta-kaoka	2021, 2016
94	3*β*-acetoxyl atractylon	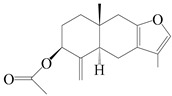	*A. macrocephala* [62]	China (Qimen county, Anhui province)	1997
95	4-ketone-atractylenolide III	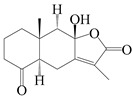	*A. macrocephala* [38]	Korea (Ulsan-si)	2016
96	13-hydroxyl-atractylenolide II	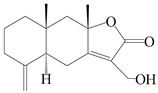	*A. macrocephala* [38]	Korea (Ulsan-si)	2016
97	Atractylenolactam	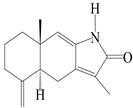	*A. macrocephala* [38]	Korea (Ulsan-si)	2016
98	Atractylon	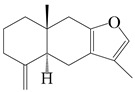	*A. macrocephala* [52], *A. lancea* [35]	China (Pan’an county, Zhejiang province); Kampo Research Laboratories, Kracie Pharma, Ltd., Takaoka	2018, 2016
99	Eudesm-4(15)-ene-7*β*,11-diol	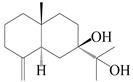	*A. macrocephala* [38]	Korea (Ulsan-si)	2016
100	Eudesma-4(14),7(11)-dien-8-one	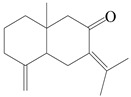	*A. lancea* [63]	Japan (Koshiro Co., Ltd.)	2017
101	Selina-4(14),7,11-trien-9-ol	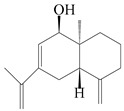	*A. macrocephala* [28]	China (Jiaozuo city, Henan province)	2022
102	Selina-4(14),11-dien-7-ol	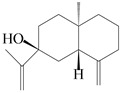	*A. macrocephala* [28]	China (Jiaozuo city, Henan province)	2022
103	Atractin A	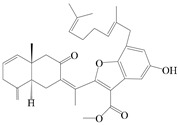	*A. macrocephala* [64]	China (Jinan city, Shandong province)	2022
104	Selina-4(14),7-dien-11-ol	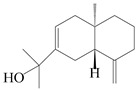	*A. macrocephala* [28]	China (Jiaozuo city, Henan province)	2022

‘–’ denotes no useful information found in the study.

### 3.2. Guaiane-Type Sesquiterpenes

The basic skeleton of guaiane sesquiterpenes contains a five-membered ring combined with a seven-membered ring, with methyl substitutions at C-1 and C-4 and an isopropyl substitution at C-7, which often forms a lactone structure. Si et al. [18] isolated two pairs of guaiane-type sesquiterpene enantiomers (**105**/**106**, and **107**/**108**) from the rhizomes of *A. macrocephala* via chiral-phase HPLC resolution. Five guaiane-type sesquiterpenes containing an interesting epoxy unit (**109**–**112**) and a rare tricyclic carbon skeleton type (**113**) were isolated from rhizomes of *A. lancea* using silica gel column chromatography and preparative HPLC, and the structures and relative configurations were determined via NMR and MS spectroscopic data [58]. Liu et al. [65] elucidated a secoguaiane lactone glycoside featuring 6/7 cores, named secoatractylohexone A (**114**), and a 9,10-unsaturated guaiene-type glycoside, named dihydroxy-9-guaine-3-one-11-*O*-*β*-d-glucopyranoside (**115**), along with three known guaiane-type sesquiterpenes (**116**–**118**), from the rhizomes of *A. lancea* on the basis of extensive spectroscopic data and the application of the CD technique. 4,10,11-trihydroxyguaiane (**119**), atrchiterpene D (**120**), and macrochaetoside B (**121**) were elucidated from *A. lancea* using NMR spectra and HRESIMS [47]. The EtOAc fraction of the *A. macrocephala* rhizomes was subjected to silica gel, Sephadex LH-20 column chromatography, and semi-preparative HPLC to obtain atractylmacrol A (**122**) [54]. A new guaiane-type sesquiterpenoid glycoside, namely (3*R*,4*S*,7*R*,10*R*)-2-hydroxypancherione-11-*O*-*β*-d-glucopyranoside (**123**), was identified from *A. lancea* using NMR, MS, and ECD data [17]. The atractyloside A (**124**), 10-epi-atractyloside A (**125**) (1*S*,4*S*,5*S*,7*R*,10*R*)-10,11,14-trihydroxyguai-3-one-11-*O*-*β*-d-glucopyranoside (**126**), (1*S*,4*S*,5*R*,7*R*,10*R*)-11,14-dihydroxyguai-3-one 11-*O*-*β*-d-glucopyranoside (**127**), atractyloside B (**128**), and (1*S*,5*R*,7*R*,10*R*)-secoatractylolactone-11-*O*-*β*-d-glucopyranoside (**129**) have been isolated and identified in *A. macrocephala* and *A. lancea* [17,42,55,66]. Phytochemical investigations of the rhizomes of *A. lancea* identified two previously described guaiane-type sesquiterpenes, namely (1*S*,4*S*,5*R*,7*R*,10*S*)-4,11,14-trihydroxyguai-3-one-11-*O*-*β*-d-glucopyranoside (**130**) and (1*S*,4*S*,5*R*,7*R*)-4,11,14-trihydroxyguaia-9-en-3-one-11-*O*-*β*-d-glucopyranoside (**131**), and the structures of the isolated compounds were elucidated using NMR spectroscopic analyses [67,68]. Three guaiane lactone glycosides have been identified and isolated from *A. lancea* via HPLC and elucidated through detailed spectroscopic methods, namely (1*R*,7*R*,10*S*)-10,11-dihydroxy-4-guaien-3-one 11-*O*-*β*-d-glucopyranoside (**132**), atractyloside A 14-*O*-*β*-d-fructofuranoside (**133**), and 1*β*,5*α*,7*α*-H-3*β*,4*α*,11,14-tetrahydroxy-guaia-9-en-11-*O*-*β*-d-glucopyranoside (**134**) [53,69]. Guai-10(14)-en-11-ol (**135**) was isolated from *A. macrocephala* rhizomes via silica gel column chromatography; its chemical structure was determined by a combination of 1D and 2D NMR analysis and mass spectrometry [43]. A new guaiane-type sesquiterpene, named seco-guaione (**136**), was recently isolated from a 95% ethanol extraction of *A. lancea* using macroporous resin, silica gel, and semi-preparative HPLC, and the chemical structure was identified via physiochemical and spectroscopic evidence [29]. The guaiane-type sesquiterpenoids from genus *Atractylodes* DC. are shown in Table 2.

**Table 2:** Guaiane-type sesquiterpenoids from genus *Atractylodes* DC.

NO.	Compounds	Structure	Source	Collection Area	Year
105	(4*S*,5*S*)-atractylmacrene A	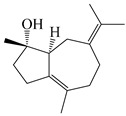	*A. macrocephala* [18]	China (Bozhou Medicinal Materials Market)	2021
106	(4*R*,5*R*)-atractylmacrene A	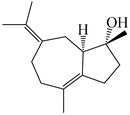	*A. macrocephala* [18]	China (Bozhou Medicinal Materials Market)	2021
107	(1*S*,4*S*,5*S*)-atractylmacrene B	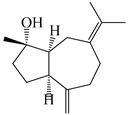	*A. macrocephala* [18]	China (Bozhou Medicinal Materials Market)	2021
108	(1*R*,4*R*,5*R*)-atractylmacrene B	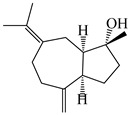	*A. macrocephala* [18]	China (Bozhou Medicinal Materials Market)	2021
109	4*α*,7*α*-epoxyguaiane-10*α*,11-diol	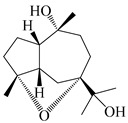	*A. lancea* [58]	China (Lanzhou city, Gansu province)	2008
110	7*α*,10*α*-epoxyguaiane-4*α*,11-diol	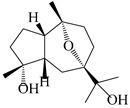	*A. lancea* [58]	China (Lanzhou city, Gansu province)	2008
111	10*β*,11*β*-epoxyguaiane-1*α*,4*α*-diol	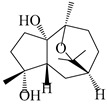	*A. lancea* [58]	China (Lanzhou city, Gansu province)	2008
112	10*β*,11*β*-epoxyguaiane-1*α*,4*α*,7*α*-triol	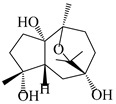	*A. lancea* [58]	China (Lanzhou city, Gansu province)	2008
113	1-Patchoulene-4*α*,7*α*-diol	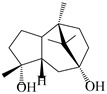	*A. lancea* [58]	China (Lanzhou city, Gansu province)	2008
114	Secoatractylohexone A	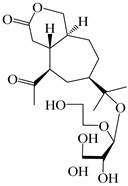	*A. lancea* [65]	China (Maoshan mountain in Jiangsu province)	2022
115	Dihydroxy-9-guaine-3-one-11-*O*-*β*-d-glucopyranoside	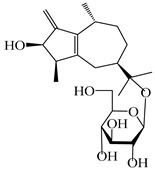	*A. lancea* [65]	China (Maoshan mountain in Jiangsu province)	2022
116	(1*S*,4*S*,5*S*,7*R*,10*S*)-10,11,14-trihydroxyguai-3-one-11-*O*-*β*-d-glucopyranoside	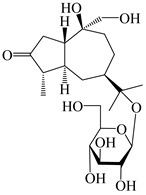	*A. lancea* [65]	China (Maoshan mountain in Jiangsu province)	2022
117	(1*S*,4*S*,5*R*,7*R*,10*R*)-11,14-dihydroxyguai-3-one-11-*O*-*β*-d-glucopyranoside	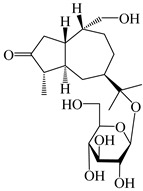	*A. lancea* [65]	China (Maoshan mountain in Jiangsu province)	2022
118	(1*S*,5*R*,7*R*,10*R*)-secoatractylolactone	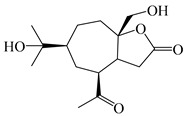	*A. macrocephala* [42], *A. lancea* [65]	Vietnam (Quan ba city, Ha Giang province), China (Maoshan mountain in Jiangsu province)	2023, 2022
119	4,10,11-trihydroxyguaiane	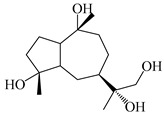	*A. lancea* [47]	China (Heilongjiang province)	2022
120	Atrchiterpene D	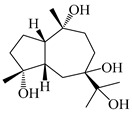	*A. lancea* [47]	China (Heilongjiang province)	2022
121	Macrochaetoside B	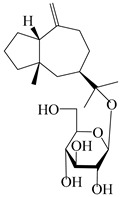	*A. lancea* [47]	China (Heilongjiang province)	2022
122	Atractylmacrol A	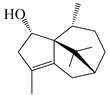	*A. macrocephala* [54]	China (Juhuacun Chinese Traditional Medicine Market, Kunming city, Yunnan province)	2018
123	(3*R*,4*R*,7*R*,10*R*)-2-hydroxypancherione-11-*O*-*β*-d-glucopyranoside	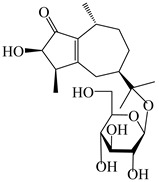	*A. lancea* [17]	China (Huanggang city, Hubei province)	2018
124	Atractyloside A	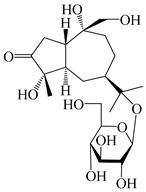	*A. lancea* [55]	Japan (Tokyo Metropolitan Medical Plants Garden)	2003
125	10-*epi*-atractyloside A	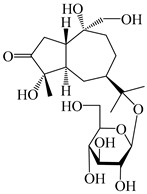	*A. lancea* [55]	Japan (Tokyo Metropolitan Medical Plants Garden)	2003
126	(1*S*,4*S*,5*S*,7*R*,10*R*)-10,11,14-trihydroxyguai-3-one-11-*O*-*β*-d-glucopyranoside	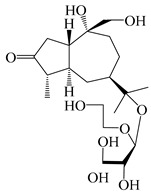	*A. lancea* [66]	Japan (Tokyo Metropolitan Medical Plants Garden)	2003
127	(1*S*,4*S*,5*R*,7*R*,10*R*)-11,14-dihydroxyguai-3-one 11-*O*-*β*-d-glucopyranoside	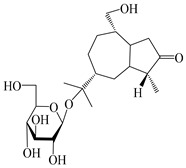	*A. lancea* [66]	Japan (Tokyo Metropolitan Medical Plants Garden)	2003
128	Atractyloside B	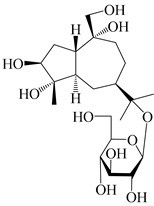	*A. lancea* [55]	Japan (Tokyo Metropolitan Medical Plants Garden)	2003
129	(1*S*,5*R*,7*R*,10*R*)-secoatractylolactone-11-*O*-*β*-d-glucopyranoside	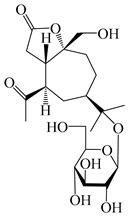	*A. macrocephala* [42], *A. lancea* [55]	Vietnam (Quan ba city, Ha Giang province); Japan (Tokyo Metropolitan Medical Plants Garden)	2023, 2003
130	(1*S*,4*S*,5*R*,7*R*,10*S*)-4,11,14-trihydroxyguai-3-one-11-*O*-*β*-d-glucopyranoside	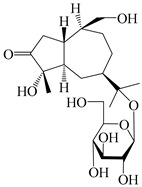	*A. lancea* [67]	China (Maoshan mountain of Jiangsu province)	2015
131	(1*S*,4*S*,5*R*,7*R*)-4,11,14-trihydroxy-guaia-9-en-3-one-11-*O*-*β*-d-glucopyranoside	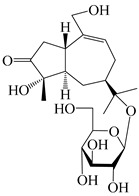	*A. lancea* [68]	China (Nanjing city, Jiangsu province)	2023
132	(1*R*,7*R*,10*S*)-10,11-dihydroxy-4-guaien-3-one 11-*O*-*β*-d-glucopyranoside	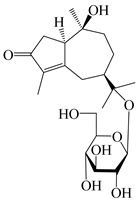	*A. lancea* [53]	China (Huanggang city, Hubei province)	2018
133	Atractyloside A 14-*O*-*β*-d-fructofuranoside	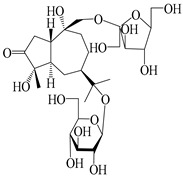	*A. lancea* [66]	Japan (Tokyo Metropolitan Medical Plants Garden)	2003
134	1*β*,5*α*,7*α*-H-3*β*,4*α*,11,14-tetrahydroxy-guaia-9-en-11-*O*-*β*-d-glucopyranoside	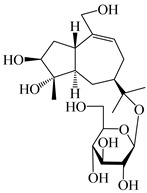	*A. lancea* [69]	China (Maoshan mountain of Jiangsu province)	2015
135	Guai-10(14)-en-11-ol	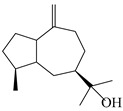	*A. macrocephala* [43]	China (Jiaozuo city, Henan province)	2022
136	Seco-guaione	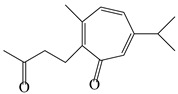	*A. lancea* [29]	China (Bozhou city, Anhui province)	2023

### 3.3. Spirovetivane-Type Sesquiterpenes

Spirovetivane-type sesquiterpenoids possess a five-membered ring and a six-membered ring connected by a spiro atom. Hinesol (**137**) was obtained from *A. lancea* using preparative silica gel column chromatography [70]. (4*R*,5*S*,7*R*)-hinesolone-11-*O*-*β*-d-glucopyranoside (**138**) and Hinesolone (**139**) were separated from the rhizomes of *A. lancea* using silica gel column chromatography [71,72]. Kamauchi et al. [48] obtained 2-oxo-hinesol (**140**), 2-oxo-12-hydroxy-hinesol (**141**), and 2-oxo-15-hydroxy-hinesol (**142**) from *A. lancea* fermented by marine fungus. Eight new spirovetivane-type sesquiterpenoids (**143**–**150**) were identified from the *n*-BuOH section of an aqueous EtOH extraction of *A. lancea* using NMR, MS, and ECD data [17]. The spirovetivane-type sesquiterpenoids from genus *Atractylodes* DC. are shown in Table 3.

**Table 3:** Spirovetivane-type sesquiterpenoids from genus *Atractylodes* DC.

NO.	Compounds	Structure	Source	Collection Area	Year
137	Hinesol	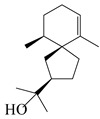	*A. lancea* [70]	Japan (Uchida Wakanyaku Ltd., Lot No. 08M1145)	2015
138	(4*R*,5*S*,7*R*)-hinesolone-11-*O*-*β*-d-glucopyranoside	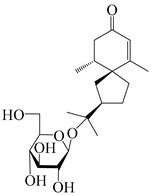	*A. lancea* [71]	China (Jurong city, Jiangsu province)	2020
139	Hinesolone	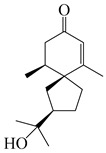	*A. lancea* [72]	China (Chinese drug store, Taipei city, Taiwan province)	2000
140	2-oxo-hinesol	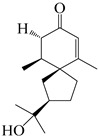	*A. lancea* [48]	Japan (Kinokuniyakanyakkyoku. Co., Ltd.)	2015
141	2-oxo-12-hydroxy-hinesol	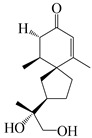	*A. lancea* [48]	Japan (Kinokuniyakanyakkyoku. Co., Ltd.)	2015
142	2-oxo-15-hydroxy-hinesol	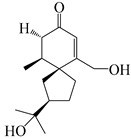	*A. lancea* [48]	Japan (Kinokuniyakanyakkyoku. Co., Ltd.)	2015
143	(7*R*)-3,4-dehydrohinesolone-11-*O*-*β*-d-glucopyranoside	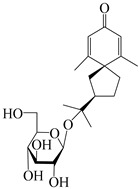	*A. lancea* [17]	China (Huanggang city, Hubei province)	2018
144	(7*R*)-3,4-dehydrohinesolone-11-*O*-*β*-d-apiofuranosyl-(1→6)-*β*-Dglucopyranoside	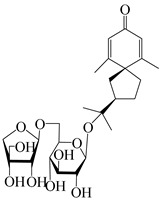	*A. lancea* [17]	China (Huanggang city, Hubei province)	2018
145	(5*R*,7*R*)-14-hydroxy-3,4-dehydrohinesolone-11-*O*-*β*-d-glucopyranoside	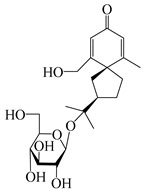	*A. lancea* [17]	China (Huanggang city, Hubei province)	2018
146	(5*R*,7*R*)-14-hydroxy-3,4-dehydrohinesolone-11-*O*-*β*-d-apiofuranosyl-(1→6)-*β*-d-glucopyranoside	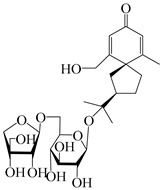	*A. lancea* [17]	China (Huanggang city, Hubei province)	2018
147	(5*R*,7*R*)-14-hydroxy-3,4-dehydrohinesolone-14-*O*-*β*-d-xylopyranoside	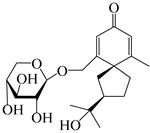	*A. lancea* [17]	China (Huanggang city, Hubei province)	2018
148	(4*R*,5*S*,7*R*)-14-hydroxyhinesolone-14-*O*-*β*-d-xylopyranoside	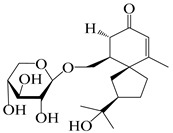	*A. lancea* [17]	China (Huanggang city, Hubei province)	2018
149	(3*S*,4*S*,5*S*,7*R*)-3-hydroxyhinesolone-11-*O*-*β*-d-glucopyranoside	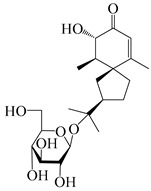	*A. lancea* [17]	China (Huanggang city, Hubei province)	2018
150	(4*S*,5*S*,7*R*)-15-hydroxyhinesolone-15-*O*-*β*-d-xylopyranoside	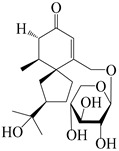	*A. lancea* [17]	China (Huanggang city, Hubei province)	2018

### 3.4. Isopterocarpolone-Type Sesquiterpenes

Isopterocarpolone-type sesquiterpenoids usually possess a 6-(2-hydroxypropan-2-yl)-4,8a-dimethyl-1,4*α*,5,6,7,8-hexahydronaphthalen-2-one skeleton. 14-hydroxy-isopterocarpolone (**151**) was identified in *A. lancea* via physiochemical and spectroscopic analyses [48]. Atractyloside I (**152**) was described in *A. lancea* [55]. Meanwhile, another *Cis*-isomerism (**153**) was also found in *A. lancea* [66]. Jiang et al. [53] reported that three isopterocarpolone-type sesquiterpenoids, (5*R*,7*R*,10*S*)-14-hydroxylisopterocarpolone-11-*O*-*β*-d-glueopyranoside (**154**), (5*R*,7*R*,10*S*)-3-*O*-*β*-d-glucopyranosylisopterocarpolone-11-*O*-*β*-d-apiofuranosyl-(1→6)-*β*-d-glucopyranoside (**155**), and (5*R*,7*R*,10*S*)-14-carboxylisopterocarpolone-11-*O*-*β*-d-glucopyranoside (**156**), were isolated from *A. lancea* using HPLC and elucidated through detailed spectroscopic methods. Five isopterocarpolone-type sesquiterpenoids (**157**–**161**) have also been identified in this species using extensive spectroscopic analyses with experimental and ECD calculations [57]. The isopterocarpolone-type sesquiterpenoids from genus *Atractylodes* DC. are shown in Table 4.

**Table 4:** Isopterocarpolone-type sesquiterpenoids from genus *Atractylodes* DC.

NO.	Compounds	Structure	Source	Collection Area	Year
151	14-hydroxy-isopterocarpolone	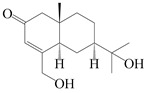	*A. lancea* [48]	Japan (Kinokuniyakanyakkyoku. Co., Ltd.)	2015
152	Atractyloside I	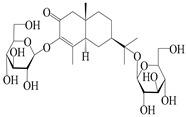	*A. lancea* [55]	Japan (Tokyo Metropolitan Medical Plants Garden)	2003
153	*Cis*-atractyloside I	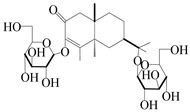	*A. lancea* [66]	Japan (Tokyo Metropolitan Medical Plants Garden)	2003
154	(5*R*,7*R*,10*S*)-14-hydroxylisopterocarpolone-11-*O*-*β*-d-glueopyranoside	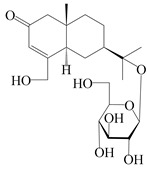	*A. lancea* [53]	China (Huanggang city, Hubei province)	2018
155	(5*R*,7*R*,10*S*)-3-*O*-*β*-d-glucopyranosylisopterocarpolone-11-*O*-*β*-d-apiofuranosyl-(1→6)-*β*-d-glucopyranoside	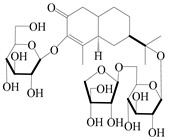	*A. lancea* [53]	China (Huanggang city, Hubei province)	2018
156	(5*R*,7*R*,10*S*)-14-carboxylisopterocarpolone-11-*O*-*β*-d-glucopyranoside	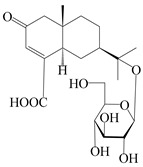	*A. lancea* [53]	China (Huanggang city, Hubei province)	2018
157	(5*R*,7*R*,10*S*)-3-hydroxylisopterocarpolone-3-O-*β*-d-glucopyranoside	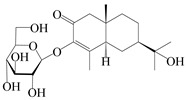	*A. lancea* [57]	China (Huanggang city, Hubei province)	2016
158	(5*R*,7*R*,10*S*)-6″-*O*-acetylatractyloside I	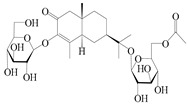	*A. lancea* [57]	China (Huanggang city, Hubei province)	2016
159	(5*R*,7*R*,10*S*)-6′-*O*-acetylatractyloside I	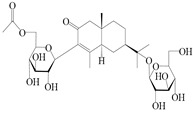	*A. lancea* [57]	China (Huanggang city, Hubei province)	2016
160	(5*R*,7*R*,10*S*)-isopterocarpolone-11-*O*-*β*-d-apiofuranosyl-(1→6)-*β*-d-glucopyranoside	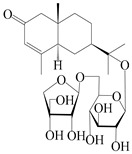	*A. lancea* [57]	China (Huanggang city, Hubei province)	2016
161	(5*R*,7*R*,10*S*)-isopterocarpolone-11-*O*-*β*-d-glucopyranoside	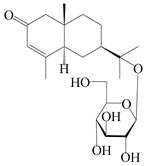	*A. lancea* [66]	Japan (Tokyo Metropolitan Medical Plants Garden)	2003

### 3.5. Eremophilane-Type Sesquiterpenes

Eremophilane-type sesquiterpenes are widely present in several genera (such as *Ligularia*, *Senecio*, and *Cacalia*) of Asteraceae [73,74]. However, this class of sesquiterpenoids shows few forms and narrow distribution in *Atractylodes* DC. species. Currently, only two eremophilane-type sesquiterpenoids, namely (3*S*,4*R*,5*R*,7*R*)-3,11-dihydroxy-11,12-dihydronootkatone-11-*O*-*β*-d-glucopyranoside (**162**) and (3*S*,4*R*,5S,7*R*)-3,4,11-trihydroxy-11,12-dihydronootkatone-11-*O*-*β*-d-glucopyranoside (**163**), have been isolated from *A. lancea* using RP-18, Sephadex LH-20 column chromatography, and semi-preparative HPLC and elucidated through NMR, HRESIMS, and ECD calculations [57]. The eremophilane-type sesquiterpenoids from genus *Atractylodes* DC. are shown in Table 5.

**Table 5:** Eremophilane-type sesquiterpenoids from genus *Atractylodes* DC.

NO.	Compounds	Structure	Source	Collection Area	Year
162	(3*S*,4*R*,5*R*,7*R*)-3,11-dihydroxy-11,12-dihydronootkatone-11-*O*-*β*-d-glucopyranoside	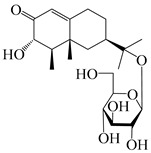	*A. lancea* [57]	China (Huanggang city, Hubei province)	2016
163	(3*S*,4*R*,5S,7*R*)-3,4,11-trihydroxy-11,12-dihydronootkatone-11-*O*-*β*-d-glucopyranoside	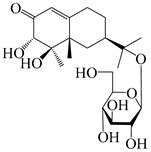	*A. lancea* [57]	China (Huanggang city, Hubei province)	2016

### 3.6. Biosynthesis of Sesquiterpenes

**Figure 1:**
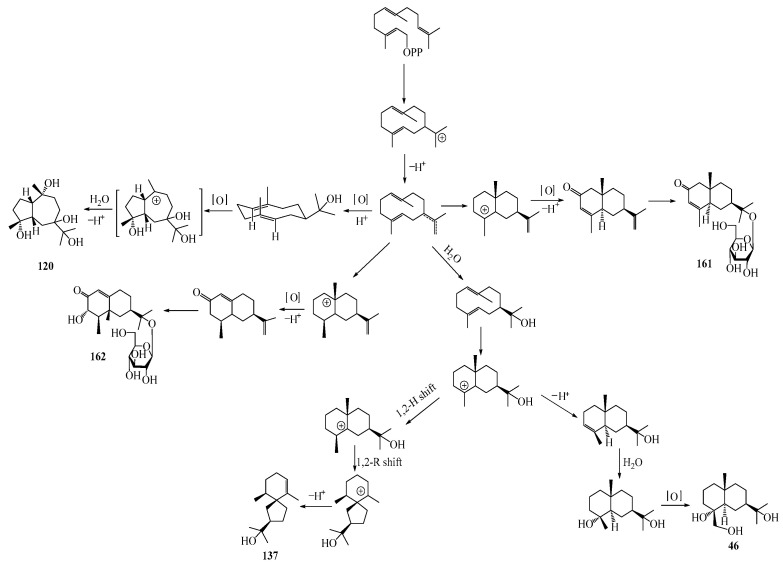
The putative biosynthetic pathways of eudesmane-type (**46**), guaiane-type (**120**), spirovetivane-type (**137**), isopterocarpolone-type (**161**), and eremophilane-type (**162**) sesquiterpenes.

Farnesyl pyrophosphate (FPP) has been recognized as a sesquiterpenoid biosynthetic precursor and generates diverse sesquiterpene carbon skeletons via irregular coupling reactions [75,76]. FPP undergoes one cyclization or more to form germacryl cations, which lose a proton to produce the intermediate germacrenes A/B, followed by a series of protonation, structural rearrangements, and substitutions of various hydroxyls via oxidation reactions to produce eudesmane, guaiane, spirovetivane, isopterocarpolone, and eremophilane skeletons [77,78,79]. The possible biosynthetic pathways of various sesquiterpene types are shown in Figure 1.

## 4. Pharmacological Activities

Pharmacological studies have shown that the majority of *Atractylodes* DC. species exhibit anticancer, anti-inflammatory, antibacterial and antiviral, antioxidant, neuroprotective, and gastrointestinal protection properties. The bioactivities and the corresponding pharmacological mechanisms of the crude extract and isolated sesquiterpenes are listed in Table 6. These findings support the traditional use of *Atractylodes* DC. in terms of pharmacological activity.

### 4.1. Anticancer Activity

Mao et al. [80] determined that an appropriate concentration of atractylon can inhibit the proliferation and promote the apoptosis of intestinal cancer cells by suppressing the PI3K/AKT/mTOR signaling pathway. In addition, atractylon regulates the expression of thymopoietin antisense transcript 1 (TMPO-AS1) and coiled-coil domain-containing 183 antisense RNA 1 (CCDC183-AS1) and inhibits the invasion and migration of liver cancer cells [81]. *β*-eudesmol was found to have moderate activity against human cholangiocarcinoma (HuCCT-1) cell growth with an IC_50_ (concentration that inhibits cell growth by 50%) value of 16.80 ± 4.41 µg/mL through the Notch signaling pathway and its upstream/downstream molecules in the CCA cell line at the gene and protein expression levels [82]. Moreover, *β*-eudesmol treatment (2.5–5 mg/kg) significantly inhibited the growth of H_22_ and S_180_ mouse tumor in vivo, which indicated that it inhibited angiogenesis via suppressing CREB activation in growth factor signaling pathway [83]. Hinesol can induces the apoptosis of human leukemia-60 (HL-60) cells through the JNK signaling pathway in HL-60 cells [70]. Furthermore, hinesol reduced cell proliferation via the arresting cell cycle at the G1 phase and induced apoptosis. Further experiments revealed that hinesol inhibited the phosphorylation of MEK and extracellular signal-regulated kinase (ERK) and downregulated the expressions of NF-κB p65 and phosphor-p65 in nuclei [84]. Atramacronoid A induced SGC-7901 cells apoptosis through the promotion of the synthesis of neutrophil elastase [44]. AT-I, AT-II, and atractylon showed the most potent antitumor activity against B16 cells, and they could also induce cell differentiation and inhibit cell migration through inactivating Ras/ERK MAPK (for AT-I and AT-II) and PI3/AKT pathways [85]. AT-I can downregulate the expression of cyclin-dependent kinases (CDK1) in ovarian cancer SK-OV-3 and ovarian carcinoma (OVCAR)-3 cells through the PI3K/AKT pathway, which leads to cell cycle arrest in the G2/M phase, and plays an important role in the proliferation inhibition of tumor cells [86]. AT-I inhibited the self-renewal capacity of gastric stem-like cells (GCSLCs) via the suppression of their sphere formation capacity and cell viability. AT-I attenuated gastric cancer stem cell (GCSC) traits partly through inactivating Notch1, leading to a reduction in the expressions of its downstream targets Hes1, Hey1, and CD44 in vitro [87]. AT-I showed significant antitumor activity on A549 and HCC827 cells in vitro and in vivo, and the possible mechanism of action may be related to apoptosis induced by AT-I via a mitochondria-mediated apoptosis pathway [88]. Ye et al. [89] demonstrated that the G1-arresting and apoptotic effects of AT-II in B16 cells involve p38 activation as well as ERK and Akt inactivation, and the cytotoxic/apoptotic effects of AT-II are potentially p53-dependent. AT-II exerted significant antitumor effects on gastric carcinoma cells by modulating the Akt/ERK signaling pathway, which upregulated the expression level of Bax but downregulated the expression levels of B-cell lymphoma-2 (Bcl-2), p-Akt, and p-ERK compared to those of the control group [90]. Codonolactone, also named AT-III, which inhibited the programming of the epithelial–mesenchymal transition (EMT) in vitro and in vivo, inhibited the motility of metastatic breast cancer cells through the downregulation of transforming growth factor (TGF)-*β* signaling, and blocked the activation of Runx2 phosphorylation [91]. AT-III can induce the apoptosis of lung carcinoma cells via inhibiting cell growth, increasing lactate dehydrogenase release, and modulating the cell cycle in human lung carcinoma A549 cells. In addition, it also inhibited the proliferation and capillary tube formation of human umbilical vein endothelial cells [92].

### 4.2. Anti-Inflammatory Activity

Lipopolysaccharides (LPS) act as prototypical endotoxins, inducing inflammation, septic shock, and death, and are commonly used for in vitro models of inflammation [143]. Nitric oxide (NO) is one of the inflammatory mediators of many organs; inhibitors of NO production may have therapeutic potential in the treatment of inflammation accompanying the overproduction of NO [144]. It was determined that the existing cyclic ether on the skeleton of sesquiterpenes is responsible for protective activity against neuroinflammation in LPS-induced BV-2 microglia [45]. AT-I displayed a potent inhibitory effect on angiogenesis through the downregulation of NO, tumor necrosis factor-α (TNF-α), interleukin (IL)-1β, IL-6, VEGF, and PlGF in chronic inflammation [93]. Jin et al. [94] reported that AT-I inhibited the LPS-induced phosphorylation of p38 and ERK mitogen-activated protein kinases (MAPKs) and showed anti-inflammatory activity in RAW264.7 cells. AT-I also inhibited the proliferation of vascular smooth muscle cells (VSMCs) induced by oxidized modified low-density lipoprotein (OXLDL). Migration contributes to antiatherosclerosis by responding to the expression of monocyte chemoattractant protein-1 (MCP-1) and by downregulating the expression of effective inflammatory mediators of the vascular inflammatory response [95]. AT-I extracted from *A. macrocephala* rhizomes effectively inhibited the increase in vascular permeability in mice caused by acetic acid and reduced cotton pellet granuloma tissue proliferation significantly, which proved that it was an active compound in acute and chronic inflammation models in mice [96]. AT-I was reported previously to act on white blood cell membranes and TLR_4_, and its anti-inflammatory activity is related to antagonizing the TLR_4_ pathway [97]. AT-I shows an anti-inflammatory effect by inhibiting TNF-α and IL-6 production. The anti-inflammatory molecular mechanism of AT-I may be associated with the inhibition of the NF-κB, ERK 1/2, and p38 signaling pathways [98]. Animal studies further demonstrated that AT-I and AT-III exert their anti-inflammatory effects by downregulating lipopolysaccharide (LPS)-induced TNF-α expression and inducible NOS (iNOS) expression. Meanwhile, AT-I showed more potent inhibition than AT-III in the production of TNF-*α* and NO in LPS-activated peritoneal macrophages [99]. Moreover, in vivo experiments revealed that AT-III could alleviate osteoarthritis by inhibiting chondrocyte senescence through reduced phosphorylation of IκB kinase (IKK) *α*/*β*, IκBα, and P65 in the NF-κB pathway [100]. Li et al. [101] discovered that atractylon significantly inhibited the ERK, JNK, and NF-κB expression induced by LPS in BV2 cells. It is suggested that atractylone is able to alleviate LPS-induced inflammatory responses through the downregulation of the ERK, JNK, and NF-κB pathways in BV2 cells. Atractylon significantly inhibited NO and prostaglandin E_2_ production, as well as inducible NO synthase and cyclooxygenase-2 expression in LPS-induced RAW 264.7 cells. Atractylon also significantly reduced the acetic acid-induced writhing response, carrageenan-induced pawedema, and hot-plate latent pain response [35]. Seo et al. [102] investigated the regulatory mechanism of *β*-eudesmol on mast cell-mediated inflammatory response; the results indicated that it inhibited the production and expression of IL-6 on phorbol 12-myristate 13-acetate and calcium ionophore A23187-stimulated human mast cells (HMCs) via suppressing the activation of p38 MAPKs and NF-κB in activated HMC-1 cells, as well as the activation of caspase-1 and expression of receptor-interacting protein-2.

### 4.3. Antimicrobial and Antiviral Activity

Previous studies have proven that the spatial arrangement of the terpenoid skeleton combined with an *α*-methylene-*γ*-lactone moiety exhibits obvious antiviral activity [145]. Atractyloside A not only possesses anti-influenza B virus infection effects in vivo and in vitro but also can regulate macrophage polarization to the M2-type, which can effectively attenuate the damage caused by influenza B virus infection [103]. Shi et al. [104] reports that atractylon has anti-influenza virus A H3N2, anti-influenza virus A H5N1 (avian influenza virus), and anti-influenza B virus effects at non-toxic concentrations. Cheng et al. [105] determined that atractylon significantly alleviated influenza A virus (IAV)-induced lung injury via regulating the Toll-like receptor 7 (TLR-7) signaling pathway and may warrant further evaluation as a possible agent for IAV treatment. The essential oil of *A. lancea* exhibited antibacterial activities against both Gram-positive and Gram-negative bacteria through the simultaneous disruption of the cell membrane [106]. The administration of *A. macrocephala* ethanol extracts (5–40 mg/mL) for 24 h remarkably inhibited the growth of *Staphylococcus aureus*, *Escherichia coli*, *Bacillus subtilis*, and *Shigella felxneri* bacteria. Meanwhile, the ethanol extracts from the above-ground portion of the plant showed greater antibacterial activity than extracts of rhizome tissues [107]. Li et al. [108] demonstrated that the essential oil of *A. lancea* had antimicrobial activity against clinical isolates of multidrug-resistant *Escherichia coli*. Wan et al. [109] discovered that *atractylodes* essential oil showed antifungal activity against *Colletotrichum* karstii, *Colletotrichum* gloeosporioides, *Colletotrichum* camelliae, *Colletotrichum* fioriniae, and *Colletotrichum* chongqingense with EC_50_ values of 0.089, 0.165, 0.108, 0.205, and 0.092 mg/mL, respectively, and had a significantly higher antifungal effect in the contact phase than that in the vapor phase (*p* < 0.05).

### 4.4. Insecticidal Activity

Sesquiterpenoids are well known as major constituents of essential oils and play important ecological roles in the plants’ interactions with pollinators and predators to adapt to the environment [146]. In previous reports, atractylon and *β*-eudesmol were toxic to fruit flies (LD_50_ = 1.63 and 2.65 μg/adult, respectively), while the crude oil of *A. lancea* had an LD_50_ value of 2.44 μg/adult [49]. *β*-eudesmol exhibited contact toxicity and ovicidal activity against *Plutella xylostella* diamondback moths [110]. Although hinesol and *β*-eudesmol expressed some repellent and contact toxicities against *Tribolium castaneum* adults (red flour beetles), they displayed a lower repellency level (*p* < 0.05) than those of N,N-Diethyl-3-methyl benzoyl amide (DEET), and their contact toxicity of them was unremarkable [111]. AT-III and atractylon were proven to possess contact and fumigant toxicities against *Dermatophagoides farinae* and *Dermatophagoides pteronyssinus* house dust mite adults using fabric-circle residual contact and vapor-phase toxicity bioassays. They were much more toxic toward house dust mite adults (*D. farinae* and *D. pteronyssinus*) than either DEET or dibutyl phthalate but slightly less active than benzyl benzoate [112]. He et al. [113] determined that the hexane-soluble phase of *A. lancea* has high lavicidal activity against *Culex pipiens pallens* Coquillett, wild *Culex pipiens molestus* Forskal, and *Aedes albopictus* Skuse, which have the potential to be developed as a novel insecticide.

### 4.5. Neuroprotective Activity

To date, sesquiterpene lactones from medicinal plants have been reported to exhibit a neuroprotective effect against glutamate-induced neurotoxicity in cultured neurons [147]. Biatractylenolide exerted a neuroprotective effect against glutamate-induced excitotoxicity via decreasing the formation of reactive oxygen species (ROS) and the activity of acetylcholinesterase (AChE) and increasing the expression of synapsin I and protein kinase C (PKC) in D-galactose-treated mice, which may have therapeutic potential in aging-related memory impairment [114]. In PC12 and SH-SY5Y cells, biatractylolide could modulate PI3K-Akt-GSK3*β*-dependent pathways to protect against glutamate-induced cell damage [115]. AT-III was shown to be able to protect phaeochromocytoma (PC) 12 cells from corticosterone-induced injury by inhibiting intracellular Ca^2+^ overloading and the mitochondrial apoptotic pathway, as well as modulating the MAPK/NF-κB inflammatory pathways, which may serve as a therapeutic agent in the treatment of depression [116]. Liu et al. [117] determined that AT-III exhibited a significant neuroprotective effect against glutamate-induced neuronal apoptosis via inhibiting the caspase signaling pathway, which markedly attenuated the caspases-3-like activity and may therefore have therapeutic potential in excitotoxicity-mediated neurological diseases. In a chronic unpredictable mild stress (CUMS) mouse model, AT-I (5–20 mg/kg) increased sucrose preference and shortened the immobility time in the forced swimming and tail suspension tests and reduced CUMS-induced decreases in serotonin and norepinephrine in the hippocampus [118]. Zhou et al. [119] found that AT-III produces antidepressant- and anxiolytic-like effects, which are related to the normalization of proinflammatory cytokine levels under chronic mild stress. AT-II may reduce the injury of neuronal HT22 cells by oxidative stress through phosphatidylinositol-3 kinase/protein kinase B [120]. In a Parkinson’s disease model, AT-I, AT-II, biepiasterolid, isoatractylenolide I, and AT-III showed a significant protective effect on MPP^+^-induced SH-SY5Y cells at 1–10 μM [121]. Lin et al. [122] determined that atractylon had a protective effect against sleep-disordered breathing (SDB)-induced nerve cell injury and cognitive dysfunction (CD) via decreasing chronic intermittent hypoxia (CIH)-induced CD and the expression of inflammatory factors in the hippocampal region by suppressing M1 microglial activation and the promotion of M2 microglial activation. Moreover, the downregulation of sirtuin 3 decreased the protective effect of atractylon against CIH-induced microglial cell injury.

### 4.6. Antioxidant Activity

The antioxidant activity of the sesquiterpene lactones has been proven by their DPPH and 2,2′-azino-bis(3-ethylbenzthiazoline-6-sulphonate) (ABTS) free radical scavenging activity, and most STLs have been reported to exert their antioxidant activity through the activation of the antioxidant response element (ARE) gene [148]. Selina-4(14),7,11-trien-9-ol and selina-4(14),7(11)-dien-8-one exhibited antioxidant activity by activating the Nrf2-ARE receptor in the Keap1-Nrf2-ARE signaling pathway. Furthermore, selina-4(14),7,11-trien-9-ol binds to Keap1 via hydrogen bonds at VAL-606, and selina-4(14),7(11)-dien-8-one binds to Keap1 via hydrogen bonds at VAL-463 and VAL-465 [19]. Atractylon was shown to inhibit carbon tetrachloride (CCl_4_)-induced cytotoxicity in primary cultured rat hepatocytes and CCl_4_-induced lipid peroxidation by rat liver microsomes [123]. Hwang et al. [124] further demonstrated that atractylon, at the concentrations of 0.01, 0.1, and 1.0 mg/mL, decreased the formation of malondialdehyde (MDA) and leakage of lactate dehydrogenase (LDH) and alanine aminotransferase (ALT) and activated the repair synthesis of DNA induced by a 30 min treatment of t-BHP (1.5 mM) in primary cultured rat hepatocytes. Xiao et al. [125] demonstrated that AT-II can markedly suppress ionizing radiation (IR) damage by promoting the expression of antioxidant factors heme oxygenase-1 (HO-1) and NAD(P)H dehydrogenase quinone oxido-reductase 1 (NQO-1), which are mediated by the nuclear factor-erythroid 2-like 2 (Nrf2) signaling pathway.

### 4.7. Activity in Gastrointestinal System

AT-I stimulates intestinal epithelial cell migration and proliferation via the polyamine-mediated Ca^2+^ signaling pathway, and it may be further developed as a promising therapeutic agent to treat diseases associated with gastrointestinal mucosal injury [126]. AT-III significantly and dose-dependently suppressed gastric ulcer formation via inhibiting matrix metalloproteinase (MMP)-2 and MMP-9 expression, decreasing the extracellular matrix (ECM) damage and preventing gastric ulcer formation [127]. Nogami et al. [128] demonstrated that *β*-eudesmol markedly inhibited ulcers in Shay rats, as well as histamine- and aspirin-induced gastric ulcers, and showed antisecretory activity on gastric acid secretion stimulated by histamine in a perfused rat stomach preparation. A remarkable antagonistic effect of *β*-eudesmol against the increased gastrointestinal movement induced by neostigmine was observed in vivo (*p* < 0.05). Improvements such as an increase in body weight and the normalization of gastrointestinal movement were observed after treatment with *β*-eudesmol in spleen-deficient mice [129]. Kimura et al. [130] further determined that an extract of *A. lancea* and *β*-eudesmol may stimulate gastric emptying or small intestinal motility by inhibiting the dopamine D_2_ receptor and 5-hydroxytryptamine 3 (HT_3_) receptor. AT-I could increase fecal water content, accelerate intestinal peristalsis, and thus improve the symptoms of constipation in rats via improving intestinal flora disturbance and increasing the content of acetic acid and propionic acid [131]. Atractyloside A improved gastrointestinal function by protecting the intestinal mucosal barrier via the inhibition of the p38 MAPK pathway [132]. Animal studies further demonstrated that the processing of *A. lancea* had more satisfactory effects than the crude in treatment of gastric ulcers. The antiulcer effects of *A. lancea* could be attributed to the anti-inflammatory properties via downregulating TNF-α, interleukin 6 (IL-6), IL-8, and prostaglandin E_2_ (PGE_2_) to the gastroprotective effects via upregulating epidermal growth factor (EGF) and trefoil factor2 (TFF2) [133]. Zhang et al. [134] investigated the effects of essential oils extracted from *A. lancea* on delayed gastric emptying, gastrointestinal hormone, and hypothalamic corticotropin-releasing factor (CRF) abnormalities induced by restraint stress in rats. The results suggested that the regulative effects of the essential oils on delayed gastric emptying are preformed mainly via inhibiting the release of central CRF and the activation of the vagal pathway, which are also involved in the release of gastrointestinal hormones such as motilin, gastrin, and somatostatin. Nakai et al. [135] discovered that an aqueous extract of *A. lancea* may improve both the delays in gastric emptying and ulcers.

### 4.8. Miscellaneous Activities

Yu et al. [136] discovered that AT-I could alleviate cerebral ischemia/reperfusion injury by reducing apoptosis and inflammatory responses through the inactivation of the nuclear factor-κB pathway. Additionally, AT-I mediated protective effects against acetaminophen-induced hepatotoxicity via the TLR4/MAPKs/NF-κB pathways, which attenuated the APAP-induced activation of TLR4, NF-κB, and MAPKs (including JNK and p38) [137]. Wang et al. [138] discovered that AT-III ameliorated bile duct ligation (BDL)-induced liver fibrosis by inhibiting the PI3K/AKT signaling pathway, as well as regulating the glutamine metabolic pathway. According to Chen et al. [139], AT-II and AT-III not only reduced agonist-induced platelet aggregation and ATP secretion, downregulated p-Akt and p-p38 MAPK levels, and inhibited platelet proliferation and clot contraction but also prolonged the time to first occlusion and prolonged bleeding. The administration of AT-I (1–300 μg/mL) or AT-III (1–300 μg/mL) to mesenchymal stem cells was found to significantly increase the expression of specific chondrogenic markers, including collagen gel aggrecan, Sox9, sonic hedgehog (Shh) and its target gene Gli-1. These effects indicate that atractylenolides may enhance chondrogenic differentiation by activating the Shh pathway [140]. The sesquiterpenoid extracted from *A. lancea* showed the inhibition of blood vessel development in zebra fish embryos, which became much more expressive with an increase in concentration. *Vegfaa* gene expression were downregulated by *β*-eudesmol at all concentrations. For zebra fish embryos, *β*-eudesmol and atractylodin were lethal, showing the antiangiogenic property of *A. lancea* extracts [141]. Tsuneki et al. [142] determined that *β*-eudesmol significantly inhibited angiogenesis in subcutaneously implanted Matrigel plugs in mice and in adjuvant-induced granuloma in mice through the blockade of the ERK signaling pathway.

## 5. Conclusions

The structural characteristics, biosynthetic pathways, and biological activities of sesquiterpenes from *Atractylodes* DC. species have been updated and summarized in the present review. Over 160 sesquiterpenes have been isolated and identified from the genus; among them, eudesmane-type sesquiterpenes were the main structures found in this genus, which accounted for more than 60% of the total sesquiterpenes. Meanwhile, the possible biosynthetic pathways of five categories of sesquiterpenes were also deduced in this review. In addition, improving pharmacological mechanisms support the traditional use of *Atractylodes* DC. Nevertheless, more research is needed in this field as current studies are still insufficient, and further exploration is required for future advancements. The primary focus of research on *Atractylodes* DC. species has been directed toward *A. lancea* and *A. macrocephala*, with little attention given to other members of the genus; however, it is worth noting that these overlooked species also possess significant value in terms of their active chemical components, making them a valuable addition to *Atractylodes* DC. resources. The mechanisms of their pharmacological activities, especially their antibacterial and antiviral activity, have not yet been clarified. Atractylon, at an appropriate concentration, can significantly inhibit the proliferation and promote the apoptosis of intestinal cancer cells via suppressing the PI3K/AKT/mTOR signaling pathway, which may be a potential candidate for the treatment of colorectal cancer and other related diseases. An additional investigation is warranted to delve into the therapeutic effectiveness, potential toxicity, and safety profiles of the active components, as well as to elucidate the correlation between chemical structure and biological activity, and to assess their practical use in clinical settings.

## Figures and Tables

**Table 6:** Pharmacological activities of sesquiterpenoids from genus *Atractylodes* DC.

Pharmacology	Compounds/Extracts	Active Concentration	Experimental Model	Mechanisms/Effects	Ref.
Anticancer activity	Atractylon (**98**)	30 mg/mL	Intestinal cancer cells	Suppressing the PI3K/AKT/mTOR signaling pathway.	[80]
	Atractylon (**98**)	20 μΜ and 10 mg/kg	HepG2 liver cancer cells and BAL B/c nude mice	Regulating the expression of TMPO-AS1 and A coiled-coil domain-containing CCDC183-AS1 and subsequently inhibited the invasion and migration of hepatic carcinoma cells in vitro.	[81]
	*β*-eudesmol (**55**)	IC_50_ = 16.80 ± 4.41 µg/mL	HuCCT-1 cell	Upstream/downstream molecules in the CCA cell line at the gene and protein expression levels through the Notch signaling pathway.	[82]
	Hinesol (**137**)	100 μΜ	HL-60 cells	Inducing apoptosis through the JNK signaling pathway.	[70]
	Atramacronoid A (**18**)	13 μmol/L	SGC-7901 cells	Promoting the synthesis of NE.	[44]
	*β*-eudesmol (**55**)	10–100 μM and 2.5–5 mg/kg	HeLa SGC-7901 and BEL-7402 in vitro and H22 and S180 mice in vivo	Suppressing CREB activation in growth factor signalling pathway.	[83]
	Hinesol (**137**)	2 and 8 μg/mL	A549 and NCI-H1299 cell lines	Downregulating MEK/ERK and NF-κB pathways.	[84]
	AT-I (**1**), AT-II (**2**), and atractylenolactam (**97**)	76.46, 84.02, and 54.88 μΜ	B16 cells	Inactivating Ras/ERK MAPK (for AT-I and AT-II) and PI3/AKT pathways.	[85]
	AT-I (**1**)	20 μM	Ovarian cancer SK-OV-3 and OVCAR-3 cells	Downregulating the expression of CDK1 through PI3K/AKT pathway.	[86]
	AT-I (**1**)	0–100 μM	HGC-27, MGC-803 and MKN-45 gastric stem-like cells	Inactivating the Notch1 pathway, leading to reduced expressions of downstream target Hes1, Hey1, and CD44 in vitro.	[87]
	AT-I (**1**)	40 μM and 40 mg/kg	A549 and HCC827 cells and transplanted tumor nude mice	Inducing apoptosis via a mitochondria-mediated apoptosis pathway.	[88]
	AT-II (**2**)	75 μM	B16 cells	Increasing the expression of phosphorylated-p38, decreasing the expression of phosphorylated-Akt and phosphorylated-ERK.	[89]
	AT-II (**2**)	200 μM	HGC-27 and AGS gastric carcinoma cells	Modulating Akt/ERK signaling pathway, which upregulated the expression level of Bax but downregulated the expression levels of Bcl-2, p-Akt, and p-ERK.	[90]
	AT-III (**3**)	40 μΜ and 75 mg/kg	Human breast cancer MDA-MB-468 and MDA-MB-231 cells and Five- to six-week-old female NOD/SCID mice	Downregulating TGF-*β* signaling and blocking the activation of Runx2 phosphorylation.	[91]
	AT-III (**3**)	1–100 μM	Human lung carcinoma A549 cells	Inhibiting cell growth, increasing lactate dehydrogenase release and modulated cell cycle.	[92]
Anti-inflammatory activity	AT-I (**1**)	15.15 mg/kg and 3.89 μg/mL	FCA-induced air pouch mice and a model of the mice aortic ring co-cultured with peritoneal macrophages	Downregulating the actions of NO, TNF-α, IL-1β, IL-6, VEGF, and PlGF in chronic inflammation.	[93]
	AT-I (**1**)	IC_50_ = 5.40 ± 0.50 μM and IC_50_ = 4.41 ± 0.45 μM	RAW264.7 cells and BV2 microglial cells	Inhibiting the LPS-induced phosphorylation of p38 and ERK MAPKs.	[94]
	AT-I (**1**)	50 μM	Vascular smooth muscle cells	Responding to the expression of MCP-1 and downregulating the expression of effective inflammatory mediators of the vascular inflammatory response.	[95]
	AT-I (**1**)	300 mg/kg	Acute and chronic inflammation models in mice	Acting on white blood cell membrane and its receptors.	[96]
	AT-I (**1**)	300 mg/kg	A model of white blood cell membrane chromatography in vitro	Antagonizing TLR4 pathway.	[97]
	AT-I (**1**)	1–100 µM	RAW264.7 cells	Inhibiting of the NF-κB, ERK ½, and p38 signaling pathways.	[98]
	AT-I (**1**) and AT-III (**3**)	67.3 and 76.1 µM	Male inbred BALB/c mice	Downregulating LPS-induced TNF-α expression and iNOS expression.	[99]
	AT-III (**3**)	5 mg/kg	Osteoarthritis rat model	Reducing the phosphorylation of IKK α/β, IκBα and P65 in NF-κB pathway, as well as nuclear translocation of p65.	[100]
	Atractylon (**98**)	160 and 320 μM	BV2 cells	Downregulating the ERK, c-JNK and NF-κB pathways.	[101]
	Atractylon (**98**)	40 mg/kg	RAW 264.7cells	Inhibiting NO and prostaglandin E2 production as well as inducible NO synthase and cyclooxygenase-2 expression.	[35]
	*β*-eudesmol (**55**)	0.2–20 µM	HMC-1 cells	Suppressing the activation of p38 MAPKs and nuclear factor-κB. Suppressing the activation of caspase-1 and expression of receptor-interacting protein-2.	[102]
Antimicrobial and antiviral activity	Atractyloside A (**124**)	30 and 100 µM, 30 mg/kg	Human lung cancer cell line A549 and the canine kidney cell line MDCK, and MDCK, C57BL/6 mice	Regulating macrophage polarization to the M2-type.	[103]
	Atractylon (**98**)	78.125 μg/mL	Influenza virus A H3N2, influenza virus A H5N1, and influenza B virus	Exhibiting antivirus effect at nontoxic concentration.	[104]
	Atractylon (**98**)	10–40 mg/kg	IAV-infected mice	Activating TLR-7 pathway to induce type I IFN production and NF-κB p65 inhibition.	[105]
	*A. lancea* rhizomes essential oil, mainly composed of *β*-eudesmol (36.5%), hinesol (29.4%), elemol (4.21%), and atractylone (4.10%)	The MICs of the tested bacteria were 64, 32, 64, 32, 64, and 64 μg/mL. The MBCs were 64, 64, 128, 64, 128, and 128 μg/mL	*Staphylococcus aureus* ATCC 25923, Bacillus cereus ATCC 14579, Bacillus subtilis ATCC 6633, *Escherichia coli* ATCC 25922, *Proteus vulgaris* ATCC 12453, and *Pseudomonas aeruginosa* ATCC 27853	Disrupting the cell membrane.	[106]
	The petroleum ether extracts of *A. macrocephala* rhizomes, mainly composed of 3, 6-dimethyl-5- (prop-1-en-2-yl)-6-vinyl-4, 5, 6, 7-tetrahydrobenzofuran (72.49%) and Guaia-3, 9-diene (7.12%)	The MICs of the tested bacteria were 20, 10, 40, and 20 mg/mL. The MBCs were all >40 mg/mL	*Staphylococcus aureus*, *Escherichia coli*, *Bacillus subtilis,* and *Shigella felxneri*	Inhibiting bacterial growth.	[107]
	*A. lancea* rhizomes essential oil	2.5–25 mg/mL	Multidrug-resistant *Escherichia coli*	Showing antibacterial effect on drug-resistant bacteria.	[108]
	*Atractylodes* essential oil	The EC_50_ values of the tested bacteria were 0.089, 0.165, 0.108, 0.205, and 0.092 mg/mL	*Colletotrichum karstii*, *Colletotrichum gloeosporioides*, *Colletotrichum camelliae, Colletotrichum fioriniae*, and *Colletotrichum chongqingense*	Influencing the morphology of conidia and hyphae, biological activity of TP, MDA, SOD, AKP, and CAT, and gene expression.	[109]
Insecticidal activity	The essential oil of *A. lancea*, and atractylon (**98**) and *β*-eudesmol (**55**)	LD_50_ = 2.44 μg/adult and LD_50_ = 1.63 and LD_50_ = 2.65 μg/adult, control (commercial botanical, rotenone with an LD_50_ = 3.70 μg/adult	*Drosophila melanogaster*	Showing pronounced contact toxicity.	[49]
	*β*-eudesmol (**55**)	–	*Plutella xylostella* diamondback moth	Exhibiting antipest activity.	[110]
	*β*-eudesmol (**55**)	60.74 μg/adult	*Tribolium castaneum* adults (red flour beetles)	Possessing contact toxicity.	[111]
	AT-III (**3**) and atractylon (**98**)	103.3 and 136.2 mg/m^2^, 73.8 and 72.1 mg/m^2^	*Dermatophagoides farinae* and *Dermatophagoides pteronyssinus*	Showing acaricidal activity in the vapor phase.	[112]
	The hexane-soluble phase of *A. lancea* rhizomes	LC_50_ = 16.87 μg/mL	*Aedes albopictus*	Showing high lavicidal activity against susceptible *A. albopictus*.	[113]
Activities on the nervous system	Biatractylenolide (**88**)	1 and 2 mg/kg	D-galactose-treated mice	Decreasing the formation of ROS and the activity of AChE and increasing the expression of synapsin I and PKC.	[114]
	Biatractylolide (**88**)	8.5 mM and 10 mM	PC 12 and SH-SY5Y Cells	Modulating PI3K-Akt-GSK3*β*-dependent pathways.	[115]
	AT-III (**3**)	1–20 μmol/L	PC 12 cells	Inhibiting the intracellular Ca^2+^ overloading, inhibiting the mitochondrial apoptotic pathway, and modulating the MAPK/NF-κB inflammatory pathways.	[116]
	AT-III (**3**)	40 μM	Cerebral cortical neurons from embryos of BALB/c mice	Inhibiting caspase signaling pathway, which markedly attenuated caspases-3-like activity.	[117]
	AT-I (**1**)	5, 10, and 20 mg/kg	Chronic unpredictable mild stress mice	Inhibiting NLRP3 inflammasome activation to decrease IL-1*β* production.	[118]
	AT-III (**3**)	3, 10, and 30 mg/kg	Rat depression models	Decreasing the proinflammatory cytokines levels in the hippocampus of CUMS exposed rats.	[119]
	AT-II (**2**)	30, 40, and 50 μmol/L	Neuronal HT22 cells	Reducing the injury of neuronal HT22 cells through PI3K/AKT pathway.	[120]
	AT-I (**1**), biepiasterolid (**86**), isoatractylenolide I (**89**), and AT-III (**3**)	10 μM	SH-SY5Y cells	Playing a significant protective effect on MPP^+^-induced SH-SY5Y cells.	[121]
	Atractylon (**98**)	25 mg/kg and 25 μg/mL	Chronic intermittent hypoxia-exposed mice and CIH-induced BV2 cells	Suppressing M1 microglial activation and promoting M2 microglial activation, promoting sirtuin 3 expression.	[122]
Antioxidant activity	Selina-4(**14**),7(**11**)-dien-8-one (**100**) and selina-4(**14)**,7,11-trien-9-ol (**101**)	34.0 μM	HEK293T cells	Activating the Nrf2-ARE receptor in Keap1-Nrf2-ARE signaling pathway.	[28]
	Atractylon (**98**)	1.0 mg/mL	Wistar strain rats	Scavenging CCl_3_ radical in the absence of PBN, inhibiting lipid peroxidation by CCl_4_, and suppressing CCl4-induced liver lesion.	[123]
	Atractylon (**98**)	0.01, 0.1, 1 mg/mL	Primary cultured rat hepatocytes	Decreasing the formation of MDA and leakage of LDH and alanine ALT and repair synthesis of DNA.	[124]
	AT-II (**2**)	50 μM	HaCaT cells	Promoting the expression of antioxidant factors HO-1 and NQO-1, which are mediated by Nrf2 signaling pathway, upregulating the expression of MAPKp38.	[125]
Activity in gastrointestinal system	AT-I (**1**)	5 and 10 μM	The IEC-6 cell line	Stimulating intestinal epithelial cell migration and proliferation via the polyamine-mediated Ca^2+^ signaling pathway.	[126]
	AT-III (**2**)	0.27 mM and 10 mg/kg	Ethanol-induced PRGM cell damage in vitro and ethanol-induced acute rat gastric ulcer models in vivo	Inhibiting MMP-2 and MMP-9 expression, decreasing the ECM damage and preventing gastric ulcer formation.	[127]
	*β*-eudesmol (**55**) and hinesol (**137**)	50 and 100 mg/kg	Pylorus-ligated rat	Blocking the histamine H_2_-receptor.	[128]
	*β*-eudesmol (**55**)	60 and 120 mg/kg	Spleen-deficient mice	Exhibiting antagonistic effect of gastrointestinal movement induced by neostigmine.	[129]
	*β*-eudesmol (**55**)	100 mg/kg	Male ICR mice	Inhibiting the dopamine D_2_ receptor and HT_3_ receptor.	[130]
	AT-I (**1**)	10 mg/kg	Constipation rats	Improving intestinal flora disturbance and increasing the content of acetic acid and propionic acid.	[131]
	Atractyloside A (**124**)	1.25, 2.5, and 5 mg/kg,	Spleen-deficiency syndrome rats	Protecting the intestinal mucosal barrier via inhibition of the p38 MAPK pathway.	[132]
	*A. lancea* processed	0.625, 1.25, and 2.5 g/kg	The rat model of gastric ulcer induced by acetic acid	Downregulating TNF-α, IL-6, IL-8, and PGE_2_, upregulating EGF and TFF2.	[133]
	*A. lancea* rhizome essential oil, mainly containing *β*-eudesmol (34.15%, *w*/*w*) and hinesol (4.32%, *w*/*w*)	30, 60, and 120 mg/kg	Vagotomized rats	Inhibiting the release of central CRF and activation of vagal pathway.	[134]
	The lipophilic fractions of *A. lancea* rhizomes	4 mg/kg	Gastric emptying of rats	Improving delayed gastric emptying.	[135]
Miscellaneous activities					
Alleviate cerebral ischemia/reperfusion injury	AT-I (**1**)	50 mg/kg	Induction of middle cerebral artery occlusion in C57BL/6 mice	Inactivating the nuclear factor-κB pathway.	[136]
Ameliorate liver injury	AT-I (**1**)	60 and 120 mg/kg	C57BL/6 mice	Regulating the TLR4/MAPK/ NF-κB signaling pathways.	[137]
	AT-III (**3**)	10 and 50 mg/kg	A bile duct ligation mice model	Downregulating the activity of glutamine and glutamine metabolism.	[138]
Inhibit platelet activation	AT-II (**2**) and AT-III (**3**)	60 mg/kg	Iron chloride-induced carotid artery thrombosis mice	Reducing agonistinduced platelet aggregation and ATP secretion, downregulating p-Akt and p-p38 MAPK levels, and inhibiting platelet proliferation and clot contraction but also prolonged the time to first occlusion and prolonged bleeding.	[139]
Enhance chondrogenic differentiation	AT-I (**1**) and AT-III (**3**)	1–300 μg/mL	mesenchymal stem cells	Activating the Shh pathway.	[140]
Antiangiogenic activity	*β*-eudesmol (**55**)	6.3, 12.5, and 25 μM	Ebra fish embryos	Downregulating Vegfaa gene expression.	[141]
Block angiogenesis	*β*-eudesmol (**55**)	50–100 μM, 0.90 μmol/kg	Cerebral and peripheral vascular endothelial cells in vitro and Matrigel plugs and adjuvant-induced granuloma mice in vivo	Blockading the ERK signaling pathway.	[142]

## Data Availability

The data presented in this study are available on request from the corresponding author.
